# In Vitro Polyploidy Induction of Longshan *Lilium lancifolium* from Regenerated Shoots and Morphological and Molecular Characterization

**DOI:** 10.3390/plants14131987

**Published:** 2025-06-29

**Authors:** Yu-Qin Tang, Hong Zhang, Qin Qian, Shi-Yuan Cheng, Xiu-Xian Lu, Xiao-Yu Liu, Guo-Qiang Han, Yong-Yao Fu

**Affiliations:** 1 School of Advanced Agriculture and Bioengineering, Yangtze Normal University, Chongqing 408100, China; 2 College of Rural Revitalization, Fuyang Institute of Technology, Fuyang 236031, China

**Keywords:** *Lilium lancifolium* Thunb., polyploidization, shoot regeneration, flow cytometry, ISSR marker

## Abstract

Longshan *Lilium lancifolium* is a well-known medicinal and edible lily and has been registered as a geographical indicator in China. Polyploidization confers many advantages in lily production; however, characteristics of Longshan *L. lancifolium* improved by polyploidization have not been reported. Here, polyploidization was induced in regenerated Longshan *L. lancifolium* shoots using colchicine, and the mutant plantlets were characterized by morphological observation, flow cytometry, and inter simple sequence repeat (ISSR) marker technology. The optimal medium for inducing shoot regeneration was Murashige and Skoog (MS) media supplemented with 0.2 mg/L of naphthaleneacetic acid (NAA) and 0.4 mg/L of thidiazuron (TDZ). The greatest mutation induction effect was obtained after soaking the regenerated shoots in 0.10% colchicine for 48 h, for an 80.00% frequency of morphological variants. Forty-one mutant plantlets were subjected to flow cytometry, identifying one homozygous polyploid, ‘JD-12’, and one chimeric polyploid, ‘JD-37’. Additionally, 68 chromosomes were found in the ‘JD-12’ root tip cells. Compared with the control, both the tissue-cultured and field-generated ‘JD-12’ plantlets presented a slight decrease in plant height, a darker green leaf color, a rougher leaf surface, and a larger bulblet diameter; furthermore, the upper epidermal and guard cells of ‘JD-12’ were much larger with a significantly lower stomatal density. The ISSR marker detection indicated a genetic variation rate of 6.10% in ‘JD-12’. These results provide a basis for lily polyploidization breeding and the cultivation of superior Longshan *L. lancifolium* via shoot regeneration.

## 1. Introduction

*Lilium lancifolium* Thunb., commonly known as the tiger or Yixing lily, is an herbaceous perennial plant of the genus *Lilium* (Liliaceae). It has been named ‘juandan’ owing to its orange–red petals that curl back [1]. *L. lancifolium* is a medicinal lily and one of the three main food lilies in China. It is also widely used as a bulbous flower in landscaping and has important ornamental and economic value [2,3]. In the natural world, *L. lancifolium* is the oldest cultivated species in the genus *Lilium*; it mainly exists in a triploid form, but its origin is still unknown [4]. *L. lancifolium* has desirable traits, such as its strong growth and good resistance to the cold, drought, and *Fusarium* fungi, and is widely cultivated in the Yangtze River Basin of China. Notably, the lilies in Yixing city, Jiangsu Province and Longshan County, Hunan Province, are the most famous and are called the Yixing lily and the Longshan lily on the market [5,6].

Longshan County is the main production area of *L. lancifolium* in Hunan Province. The planting area is approximately 6667 hectares, and the total yield is 100,000 tons, accounting for approximately 70% of the total lily yield of Hunan Province. The total value of these lilies is CNY 1 billion; therefore, *L. lancifolium* is known as the “Hometown of the Chinese Lily” [6,7]. Additionally, Longshan County is a research base for lily planting standardization by the National Ministry of Science and Technology. Longshan *L. lancifolium* has been approved for registration as a national geographical indicator trademark in China. Its bulbs are rich in crude polysaccharides, organic selenium, colchicine, total saponins, total phenols, flavonoids, and total flavanols. *L. lancifolium* has the effects of nourishing the yin, clearing heat, moistening the lungs, and stopping coughs and has shown antioxidant and inhibitory effects on lung cancer cells [8,9]. Longshan *L. lancifolium* has been processed by many enterprises into a lily herbal tea, lily polysaccharides, lily noodles, lily masks, and lily wine, which are of important economic value [6]. In recent years, many Longshan *L. lancifolium* plants have been introduced to the Chongqing area and have become the main lily variety in Chongqing city. However, Longshan *L. lancifolium* production depends mainly on its asexual reproduction, such as bulb division or cutting, such that there is a relatively large number of occurrences of virus infections. This variety of lily has been degraded, especially as shown by the multi-head phenomenon in a bulb, and its quality has gradually declined due to long-term cultivation [10,11,12]. To improve the quality, Hu et al. (2022) [13] treated the bulbs of Longshan *L. lancifolium* with ^60^Co-γ rays combined with colchicine and reported that the suitable irradiation dose was from 2 to 3 Gy; however, a high-quality germplasm with a strong resistance was not obtained. At present, cultivating a new germplasm of Longshan *L. lancifolium* with desirable agronomic traits, such as a high quality and strong disease (virus) resistance, has become an important topic in the development of the lily industry.

In addition to the general characteristics of polyploids, such as gigantism, increased adaptability, and improved ornamental value, the cultivation of lily polyploids will be of great interest for improving lily breeding and selecting new varieties [14,15]. In recent decades, the induction of polyploidy in lilies has shown excellent results through the use of colchicine, ammonium thiosulfonate, or other chemical mutagens. For example, small polyploid *L*. *callosum* bulblets were obtained in vitro after a colchicine treatment [16]. Additionally, tetraploid plantlets of *L. pumilum* and *L. davidii* var. *unicolor* were induced from somatic embryos [17]. Using seeds as materials, Wang et al. (2019) [18] obtained polyploid *L. concolor* seedlings after a colchicine treatment. Triploid *L. davidii* var. *unicolor* was also cultivated from small bulblets in vitro by colchicine induction [19]. However, there have been no reports on the polyploid breeding of Longshan *L. lancifolium* until now.

Our research group has been breeding *L. lancifolium* since 2010, and, to date, the improvements in *L. lancifolium* have focused mainly on hybrid selection [14,20]. For example, three ornamental lily varieties were chosen as male parents for crossing with *L. lancifolium* using different pollination methods, and two hybrid lines were ultimately obtained [21]. Additionally, seven lily species/varieties were crossed with *L. lancifolium* via direct pollination, but only two combinations produced hybrid seedlings [22]. *L. lancifolium* from Harbin city (JD-h) was used in our previous studies, and multiple chimeric polyploids were obtained from bulblets upon colchicine induction [23,24]. Three years later, a new mutant variety of JD-h-15 was cultivated, which has since become a normal diploid plant [25].

In this study, to obtain the *L. lancifolium* polyploidy, we chose Longshan *L. lancifolium* as the material to induce shoot regeneration on Murashige and Skoog (MS) media with different ratios of hormones, and different concentrations of colchicine were used to treat the shoots in vitro to obtain mutant plantlets. In addition, chromosome ploidy in the mutant plantlets was identified by flow cytometry (FCM) and a chromosome counting method. Compared with those of the control plantlets, the morphological characteristics of the polyploid plantlets were observed in the early stage, and an inter simple sequence repeat (ISSR) marker analysis was used to identify the genetic variations in the polyploids. Taken together, these results provide a scientific basis for breeding high-quality and resistant varieties of Longshan *L. lancifolium*.

## 2. Materials and Methods

### 2.1. Plant Material and Growth Conditions

The healthy Longshan *L. lancifolium* bulbs (14–16 cm) without pests and diseases were collected from Longshan County, Hunan Province. The outer scales were disinfected with 75% alcohol for 30 s and 0.2% mercuric chloride soak for 12 min and then used as explants for the in vitro induction of sterile plantlets according to the methods described by Yang and Song (2013) [5]. All the plantlets were stored in the plant tissue culture room of the Flower Genetic Breeding Team of Yangtze Normal University. The cultivation conditions were a 14 h day/10 h night cycle at the light intensity of approximately 5000 lx and a temperature of 23 ± 2 °C.

### 2.2. Induction of Longshan L. lancifolium Shoot Regeneration

Bulblets were obtained from the plantlets that had been cultivated for 60 days on the MS media. The outer scales of the bulblets were stripped and placed on MS media, which consisted of 4.43 g/L MS base salt, 30 g/L sucrose, and 7 g/L agar, supplemented with 0.2 mg/L naphthaleneacetic acid (NAA) and different concentrations of thidiazuron (TDZ) (0, 0.2, 0.4, 0.8, or 1.0 mg/L), respectively. And the pH of MS media was adjusted to 5.8–5.9 in the 1 L of glass container and then was sterilized at 121 °C for 20 min. Regenerated shoot development was observed every 15 days, and the regeneration rate (number of shooting scales/total scale number × 100%) was calculated after 45 days. Three replicates were performed for the shoot induction.

### 2.3. Colchicine-Induced Variations in Regenerated Shoots

The shoots that had grown for 45 days on the optimal MS media were used for colchicine induction. The upper parts of the regenerated shoots were removed, and the base was subjected to colchicine treatment. Different concentrations of colchicine solution (0.05%, 0.10%, 0.15%, and 0.20%, *w*/*v*) were prepared in 2% dimethyl sulfoxide (DMSO). The shoots were soaked in colchicine solution in the dark with oscillation for 24 h, 48 h, and 72 h according to our previous studies [19,23,26]. Afterwards, the treated shoots were washed 5 times with sterile water and then transferred on MS media supplemented with 0.2 mg/L TDZ and 0.2 mg/L NAA for cultivation and observation under a 14 h day/10 h night cycle at the light intensity of approximately 5000 lx and a temperature of 23 ± 2 °C. The data was recorded at 15 days, the frequency of morphological variants was calculated as (number of morphological variants/treated number) × 100%, and the survival frequency was calculated as (survived number/treated number) × 100%.

### 2.4. Stable Cultivation of Variant Organs

It was expected that the regenerated shoots may show variations after being soaked in different colchicine solutions, such as the leaves becoming thicker or fleshy, their color becoming deeper, or parts may have rough surfaces. These organs were initially considered suspected variant organs. These organs were accurately cut off and cultivated into MS media supplemented with 0.2 mg/L TDZ and 0.2 mg/L NAA for subculture. Variant organ growth was observed every 15 days until plantlets germinated. The suspected variant plantlets were selected and distinguished on the basis of their leaf shape, leaf color, and plant growth vigor according to previous reports [16,23].

### 2.5. Identification of Chromosome Ploidy in Mutant Plantlets

The chromosome ploidy of the variant plantlets was analyzed by FCM (NovoCyte 2040R, Aisen Biotech Co., Ltd., Hangzhou, China) on the basis of the method described by Chen et al. (2018) [27]. The chromosome ploidy of the young blades of the variant plantlets and Langshan *L. lancifolium* control were determined through cell nucleus extraction, staining with propidium iodide (PI) solution, sample detection, and fluorescence intensity comparison. The chromosome numbers in the root tip cells were subsequently analyzed by the cytological method described by Fu et al. (2022) [25]. Root tip tissues sampled at different times (from 10:00 to 12:00 in the morning, from 12:00 to 14:00 in the afternoon, and from 15:00 to 17:00 in the afternoon) on the same day were selected as the pretreatment material samples, and the chromosome number in the root tip cells of the variant plants was compared with those of the control after fixation, staining, slide preparation, and microscopic observation (Olympus BX53, Tokyo, Japan). The experiments were repeated three times.

### 2.6. Morphological Observation of Polyploid Plantlets

The polyploid and Langshan *L. lancifolium* control plantlets were subcultivated on MS media supplemented with 0.5 mg/L 6-benzylaminopurine (6-BA) and 0.2 mg/L NAA for 60 days, and the plant’s height, leaf length, width, bulblet diameter, and size of the scales were measured with a Vernier caliper. A total of 30 plantlets were used for the control and the polyploid, respectively, and two replicates were performed. The upper and lower epidermis of the blades were removed with tweezers to prepare temporary slides, and the epidermal cells, guard cells, and stomata were observed under a microscope (Olympus BX53, Tokyo, Japan). For each, 150 cells from at least three leaves were analyzed and two replicates were performed. Afterwards, both the polyploid and control plantlets were transplanted and placed in the greenhouse, and the morphological differences between them were observed after 60 days of growth. The leaf micro-morphology data were calculated as above.

### 2.7. ISSR Molecular Marker Analysis

Genomic DNA was extracted from the mutant and the control plantlets via the CTAB method. A total of 16 ISSR marker primers (series UBC and 3A, TaKaRa, Tokyo, Japan) listed in Table S1 were selected for PCR amplification according to the methods described by Fu et al. (2025) [28]. At least three separate plants were used as the template for each ISSR primer for amplification, and amplification was optimized primarily by adjusting the DNA template concentration and primer annealing temperature. The amplified products were detected by agarose gel electrophoresis and imaged with a gel imager (JIAPENG, ZF-288, China). The polymorphic band variation rate (%) was calculated as (number of polymorphic bands/total number of bands detected) × 100%.

### 2.8. Statistical Analysis

The data were analyzed using Excel 2023 (Microsoft Corporation, USA) and statistically analyzed using SPSS 27 software (IBM China Company, Ltd., Beijing, China). The results are expressed as the means ± standard errors, and Student’s *t*-test (** *p* < 0.01) or Duncan’s multiple comparison (*p* < 0.05) were used to evaluate significant differences. Each experiment consisted of two or three replicates.

## 3. Results

### 3.1. Induction of Longshan L. lancifolium Regenerated Shoots

**Figure 1:**
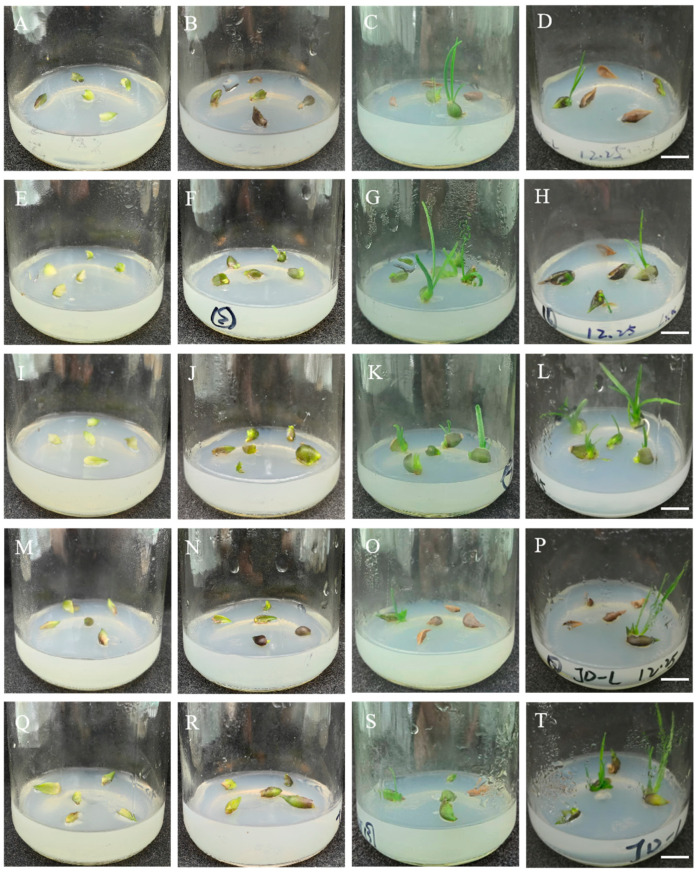
Effects of different concentrations and combinations of TDZ and NAA on the Longshan *L. lancifolium* shoot induction. (**A**–**D**): The shoot induction was performed on the MS medium with 0.2 mg/L of NAA for 0, 15, 30, and 45 days. (**E**–**H**): The shoot induction on the MS medium with 0.2 mg/L of NAA and 0.2 mg/L of TDZ for 0, 15, 30, and 45 days. (**I**–**L**): The shoot induction on the MS medium with 0.2 mg/L of NAA and 0.4 mg/L of TDZ for 0, 15, 30, and 45 days. (**M**–**P**): The shoot induction on the MS medium with 0.2 mg/L of NAA and 0.8 mg/L of TDZ for 0, 15, 30, and 45 days. (**Q**–**T**): The shoot induction on the MS medium with 0.2 mg/L of NAA and 1.0 mg/L of TDZ for 0, 15, 30, and 45 days. Bars represent 1 cm.

The outer scales of sterile bulblets were cultivated for approximately 45 days under normal light conditions. The results in Figure 1 show that some small bud points were observed on the scales after 15 days of cultivation, and the small bud morphology was notably different after 30 days. After 45 days of cultivation, some of the scales were brown and had died, and the small buds had turned into green or light-green leaves. The statistical analysis (Table S2) revealed that the shoot differentiation was greatest when the plants were cultured on the MS media supplemented with 0.2 mg/L of NAA and 0.4 mg/L of TDZ. The rate of the shoot generation from the scales was 61.11%, and the corresponding coefficient was 0.97. In contrast, the rate of the shoot generation was the lowest (17.78%) after the cultivation on the MS media supplemented with 0.2 mg/L of NAA without TDZ, and the corresponding coefficient was 0.26. Therefore, TDZ promoted the shoot differentiation of Longshan *L. lancifolium* in a dose-dependent manner.

### 3.2. Colchicine-Induced Variations in Longshan L. lancifolium Shoots

**Figure 2:**
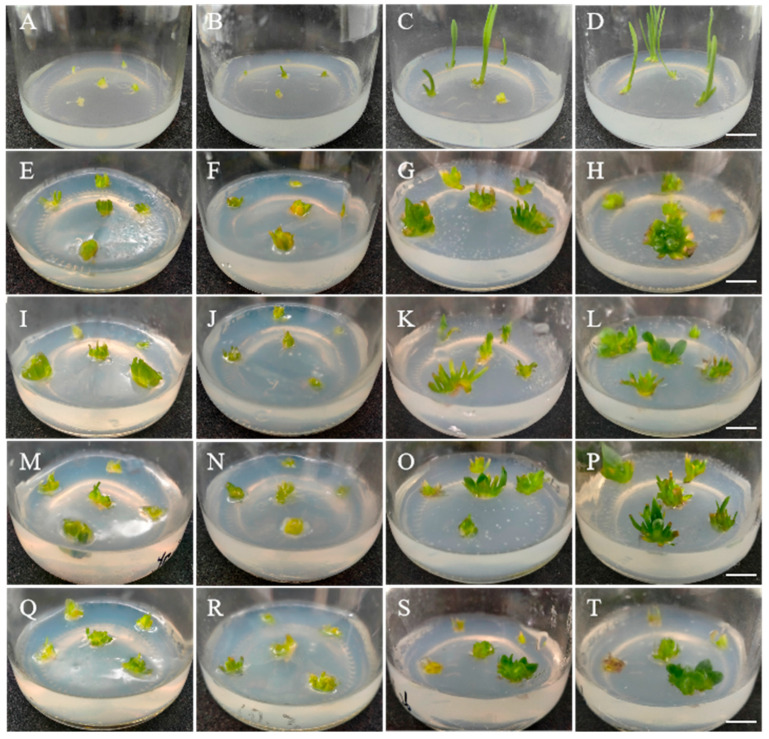
Effects of different concentrations of colchicine on the development of regenerated shoots in Longshan *L. lancifolium.* (**A**–**D**): The development of regeneration shoots in the Longshan *L. lancifolium* control for 0 d, 5 d, 10 d, and 15 d, respectively; (**E**–**H**): the development of regeneration shoots treated by 0.05% colchicine for 0 d, 5 d, 10 d, and 15 d, respectively; (**I**–**L**): the development of regeneration shoots treated by 0.10% colchicine for 0 d, 5 d, 10 d, and 15 d, respectively; (**M**–**P**): the development of regeneration shoots treated by 0.15% colchicine cultured for 0 d, 5 d, 10 d, and 15 d, respectively; (**Q**–**T**): the development of regeneration shoots treated by 0.20% colchicine cultured for 0 d, 5 d, 10 d, and 15 d, respectively; the bars represent 1 cm.

The bases of the regenerated shoots were treated with colchicine, and no significant morphological variations were observed in the regenerated shoots during the first 5 days after treatment. After 10 days, some shoots were clearly fleshy, and their color had deepened. After 15 days, the fleshiness of the shoots became more significant, and some of the unchanged shoots turned brown and died (Figure 2). As shown in Table 1, at the same concentration of colchicine (0.05–0.15%), the survival rate gradually decreased with the increasing cultivation duration, whereas the frequency of the morphological variants first increased but then decreased. Compared with 24 h or 72 h of induction, the induction for 48 h was the most suitable for inducing mutations in the regenerated shoots. In the case of the treatment for 24 h, the frequency of morphological variants was positively correlated with the concentration of colchicine. After the treatment for 48 h or 72 h, the frequency of morphological variants first increased but then decreased with the increasing colchicine concentration, and 0.10–0.15% was considered the optimal colchicine concentration for induction. Considering both the frequency of survival shoots and morphological variants, the effect of the 0.10% colchicine treatment for 48 h was the best, with an 86.67% frequency of survival shoots and an 80.00% frequency of morphological variants, followed by the 0.10% colchicine treatment for 72 h and the 0.15% colchicine treatment for 48 h, with a survival frequency of 86.67% and 83.33%, respectively, and the same frequency of 76.67% for morphological variants. Taken together, the best method for obtaining variants of Langshan *L. lancifolium* was to treat the regenerated shoots with the 0.10% colchicine for 48 h.

### 3.3. Stable Cultivation and Generation of Mutant Plantlets

**Figure 3:**
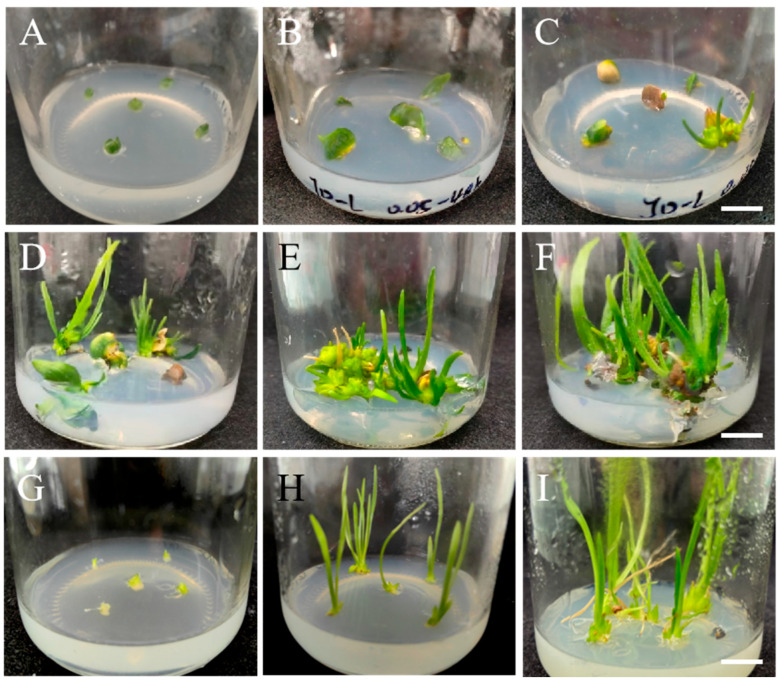
The development morphology of mutant plantlets of Longshan *L. lancifolium*. (**A**–**F**): The plant morphology of the mutant organs cultured for 0 d, 15 d, 30 d, 45 d, 60 d, and 75 d in the shoot differentiation medium, respectively. (**G**–**I**): The plant morphology of the control plantlets cultured for 0 d, 30 d, and 75 d in the shoot differentiation medium, respectively. The bars represent 1 cm of length.

The organs with obvious morphological variations were removed from the shoots and placed in MS media supplemented with 0.2 mg/L of TDZ and 0.2 mg/L of NAA for cultivation. The organ growth and development of the organs were observed, and after 15 days of culturing, the variant organs began to swell (Figure 3A,B). After 30 days of cultivation, some variant organs differentiated into novel shoots, whereas others appeared to be brown or died (Figure 3C). After 45 days of cultivation, the shoots gradually developed into leaf blades (Figure 3D). Compared with the control leaves after 60–75 days of cultivation, the variant leaves were much thicker and wider, and the leaf blades were fleshy with a deeper color and roughened surface after the same cultivation duration (Figure 3E–I). The plantlets with variant phenotypes were initially considered mutant plantlets.

### 3.4. Identification of Longshan L. lancifolium Polyploids

**Figure 4:**
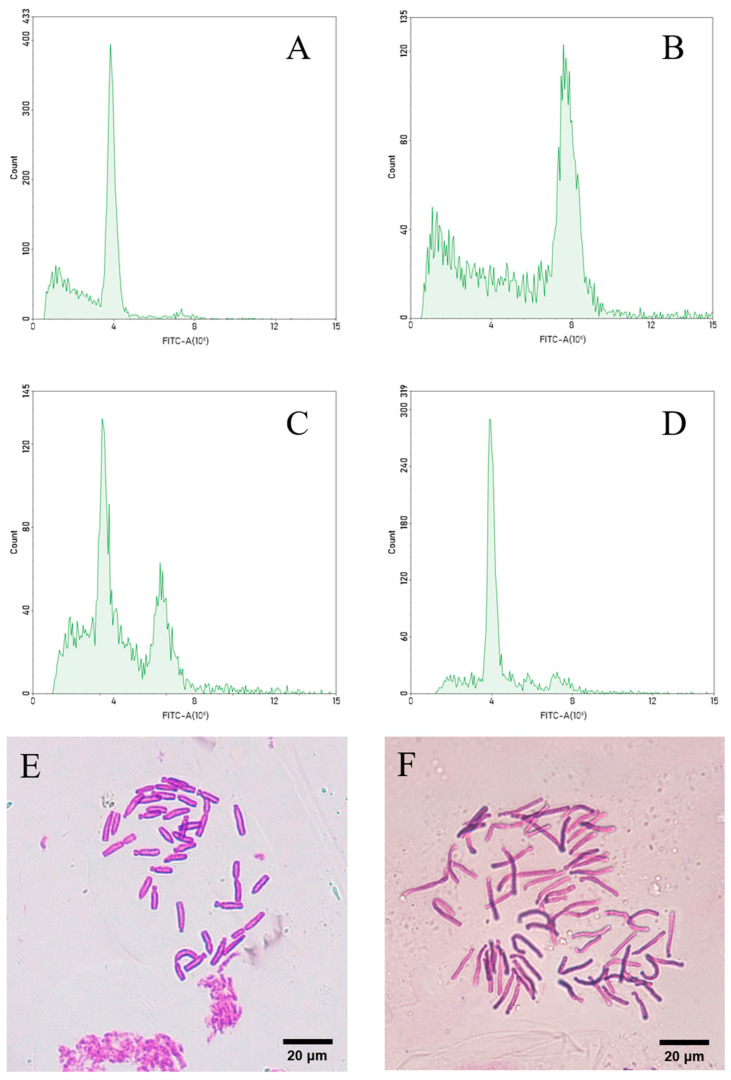
The chromosome ploidy analysis of mutant plants of Longshan *L. lancifolium*. (**A**–**D**): The analysis of the chromosome ploidy by flow cytometry; (**A**) is control plants, (**B**) is the mutant line ‘JD-12’, (**C**) is the mutant line ‘JD-37’, (**D**) represents other mutant lines, the X-axis represents the fluorescence intensity and the Y-axis represents the cell numbers; (**E**,**F**): chromosome tableting using by root tip cells, (**E**) is control plants, and (**F**) is the mutant line ‘JD-12’.

The plantlets with morphological variants were recorded, and a total of 41 plantlets with an increased leaf thickness and darker coloring were selected for the FCM analysis. Using the triploid Longshan *L. lancifolium* as a control, the chromosome ploidy was determined on the basis of the fluorescence intensity (average Mean-X). The results revealed that the peak of the triploid variety had a value of approximately four (Figure 4A). Among the 41 selected variant plantlets, No. ‘JD-12’ presented one peak at a relative fluorescence intensity value of eight, indicating that it might be a homologous polyploid (Figure 4B). No. ‘JD-37’ presented two peaks with relative fluorescence intensities of four and eight, indicating that it might be a chimera polyploid (Figure 4C). The other plants displayed only one peak with a relative fluorescence intensity value of four, which was consistent with the control, suggesting that they were triploids (Figure 4D). In addition, the total number (Y-Axis) of each line reached the basic requirement (over 2000).

### 3.5. Morphological Observation of Tissue-Cultured ‘JD-12’ Plantlets

**Figure 5:**
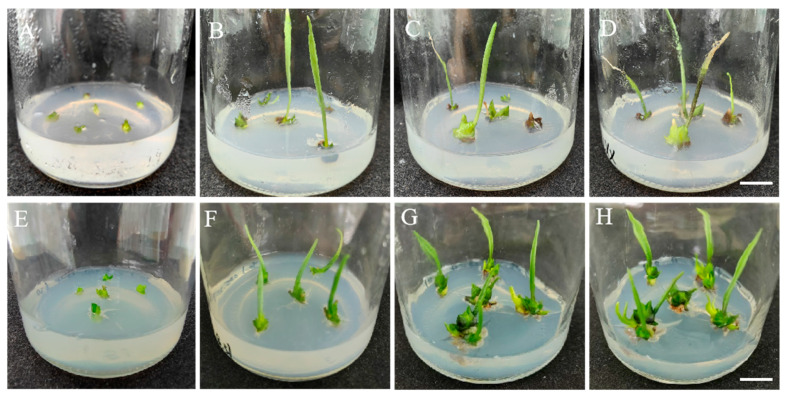
The development process of tissue-cultured ‘JD-12’ plantlets. (**A**–**D**): The development of the Longshan *L. lancifolium* control on the MS medium with 0.5 mg/L of 6-BA and 0.2 mg/L of NAA for 0 d, 21 d, 45 d, and 60 d, respectively. (**E**–**H**): The development of ‘JD-12’ on the above MS medium for 0 d, 21 d, 45 d, and 60 d, respectively. The bars represent 1 cm.

**Figure 6:**
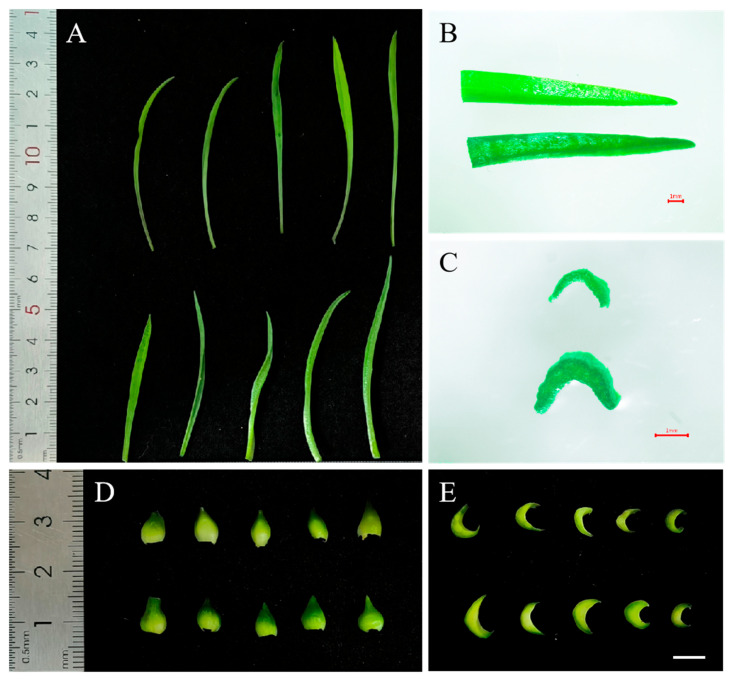
A morphological comparison of ‘JD-12’ and Longshan *L. lancifolium* plantlets. (**A**): The 60 d tissue-cultured plantlet blades in the Longshan *L. lancifolium* control (top) and ‘JD-12’ (bottom); (**B**): the upper parts in the control (top) and ‘JD-12’ (bottom) leaves; (**C**): the cross sections of the control (top) and ‘JD-12’ (bottom) leaves; (**D**): the bulblet scales in the control (top) and ‘JD-12’ (bottom); and (**E**): the cross sections of the control (top) and ‘JD-12 (bottom)’ scales. The bars represent 1 mm (**B**,**C**) and 5 mm (**E**), respectively.

‘JD-12’ and Longshan *L. lancifolium* were subcultivated for 60 days, after which their morphology was characterized (Figure 5). The results revealed that the control leaves were longer, had a smoother surface, and were flat; in contrast, the leaves of ‘JD-12’ were significantly thickened, the leaf color was deeper, and the leaf surface was rougher (Figure 6A–C). The statistical analysis (Table 2) revealed that the height of the control plantlets (7.53 cm) was slightly greater than that of the ‘JD-12’ plantlets (7.01 cm), and the leaf length (6.45 cm) and width (0.23 cm) were significantly greater than those of ‘JD-12’ (5.97 cm and 0.17 cm, respectively). Moreover, the thickness of the control leaves (0.37 mm) was significantly smaller than that of the ‘JD-12’ leaves (0.76 mm). Compared with those of the control, the average diameter of the ‘JD-12’ bulbs (0.60 cm) was significantly greater than that of the control bulbs (0.48 cm). The length and width of the outer scales were consistent between ‘JD-12’ and the control, but the scale thickness of ‘JD-12’ (2.12 mm) was significantly greater than that of the control (1.76 mm) (Figure 6D,E).

**Figure 7:**
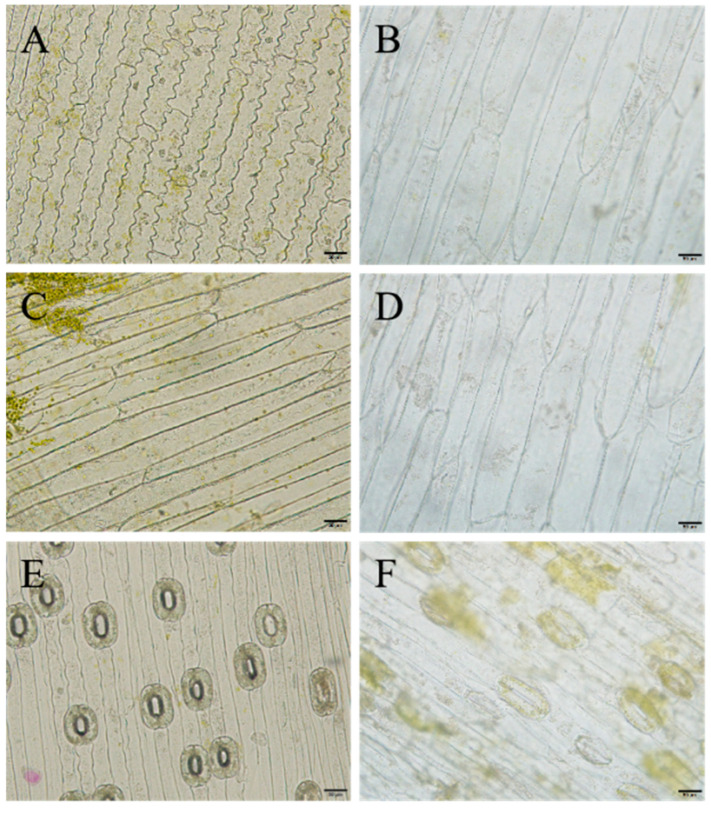
Leaf epidermal cells and guard cells in tissue-cultured ‘JD-12’ plantlets. (**A**,**C**): The upper epidermal cells of the Longshan *L. lancifolium* control. (**B**,**D**): The upper epidermal cells of ‘JD-12’. (**E**,**F**): The guard cells of the Longshan *L. lancifolium* control and ‘JD-12’, respectively. The bars represent 50 μm.

Microscopic observations of the leaf blades revealed that most of the epidermal cells of ‘JD-12’ and the control were long and had a rectangular shape, but a few were irregularly shaped. Compared with those of the control, the upper epidermal cells of ‘JD-12’ were much longer and wider and presented a greater length-to-width ratio. The transverse wall patterns of the upper epidermis were slightly wavy or straight in the control, whereas those of ‘JD-12’ were mostly straight (Figure 7). In addition, the guard cells of ‘JD-12’ were more elongated, and the stomatal density was significantly lower compared with those of the control (Table S3).

### 3.6. Phenotypic and Cellular Observations of ‘JD-12’ Plants Grown in a Greenhouse

**Figure 8:**
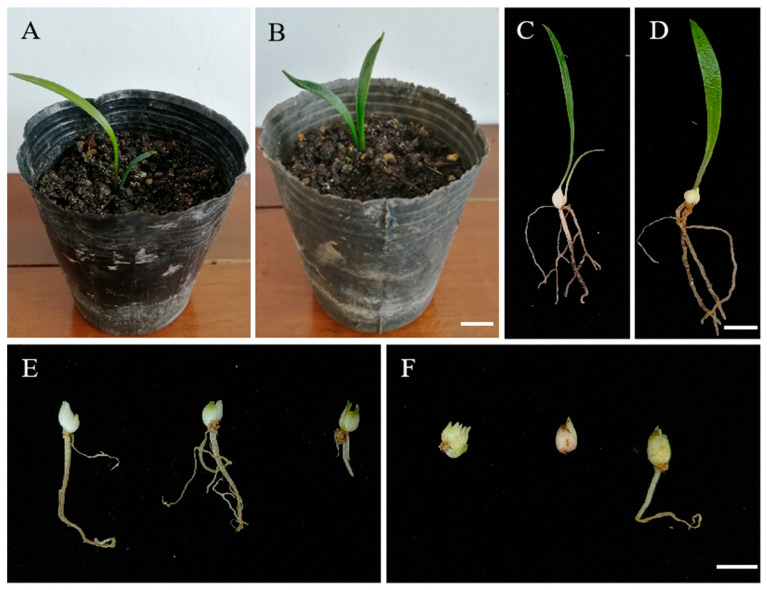
The plant morphology of ‘JD-12’ transplanting plantlets in the greenhouse. (**A**,**C**): The morphology of Longshan *L. lancifolium* control plantlets grown for 60 days in the greenhouse; (**B**,**D**): the morphology of ‘JD-12’ plantlets grown for 60 days in the greenhouse; and (**E**,**F**): the bulbs of the Longshan *L. lancifolium* control and ‘JD-12’ grown for 80 days in the greenhouse, respectively. The bars represent 1 cm.

**Figure 9:**
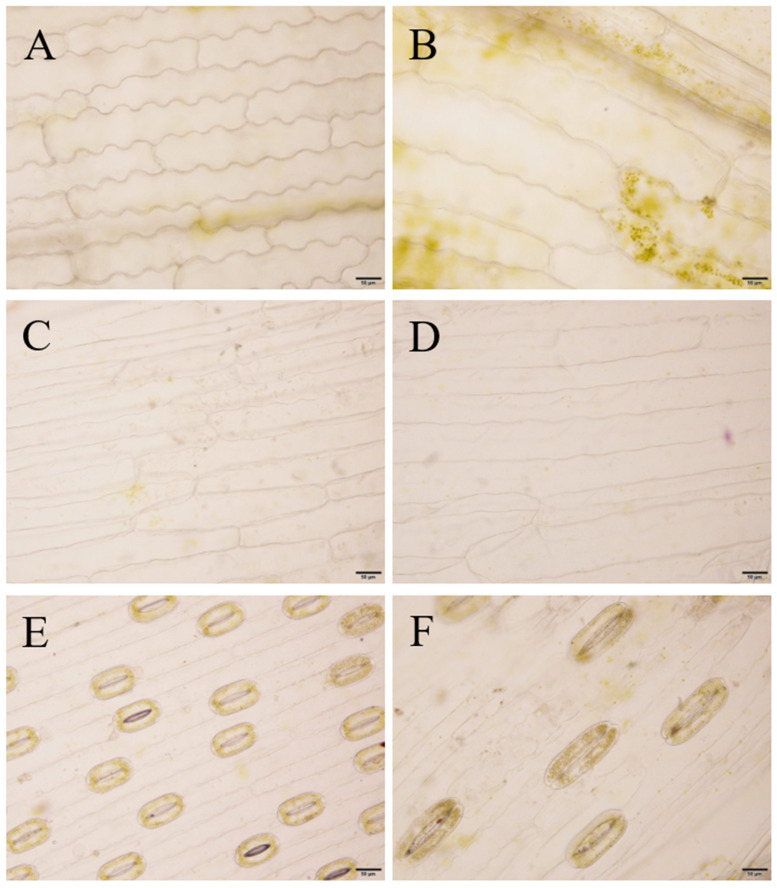
The observation of leaf epidermal cells and guard cells in JD-12 plants. (**A**,**C**): The upper epidermal cells in the control of Longshan *L. lancifolium.* (**B**,**D**): The upper epidermal cells in JD-12 plants. (**E**,**F**): The guard cells’ morphology in the control and JD-12 plants, respectively. Scale bars represent 50 μm.

The ‘JD-12’ plantlets were transplanted into a substrate mixed with peat, soil, and vermiculite at a volume ratio of 3.0:1.0:0.5 for cultivation and were observed after 60 days. As shown in Figure 8, both ‘JD-12’ and the control plants could grow normally in the greenhouse. Compared with the control, the ‘JD-12’ leaf surface was rougher, and the bulb showed a slight increase in size (Figure 8C,D). The bulb diameter was much larger (0.70 cm) in ‘JD-12’ compared with that (0.54 cm) in the control after 80 days (Figure 8E,F). These phenotypes were similar to those of the ‘JD-12’ tissue-cultured plantlets. The further observation of the leaf epidermal morphology (Figure 9, Table 3) revealed that the length and width of the upper epidermal cells in the ‘JD-12’ blades were much greater than those in the control group, with a significant difference in the cell length-to-width ratio. The ‘JD-12’ guard cells were significantly larger than the control guard cells but the stomatal density was lower, which is consistent with the morphological characteristics of the polyploids.

In addition, the root tips of ‘JD-12’ plantlets were selected for cytological evaluation, and those of Longshan *L. lancifolium* were used as controls. As shown, 68 chromosomes were present in ‘JD-12’ cells, which was significantly greater than the number (36) of chromosomes in the control cells (Figure 3E,F). Therefore, we concluded that ‘JD-12’ was a polyploid line on the basis of the FCM analysis and the root tip cytological observation.

### 3.7. ISSR Marker Detection in ‘JD-12’ Plantlets

**Figure 10:**
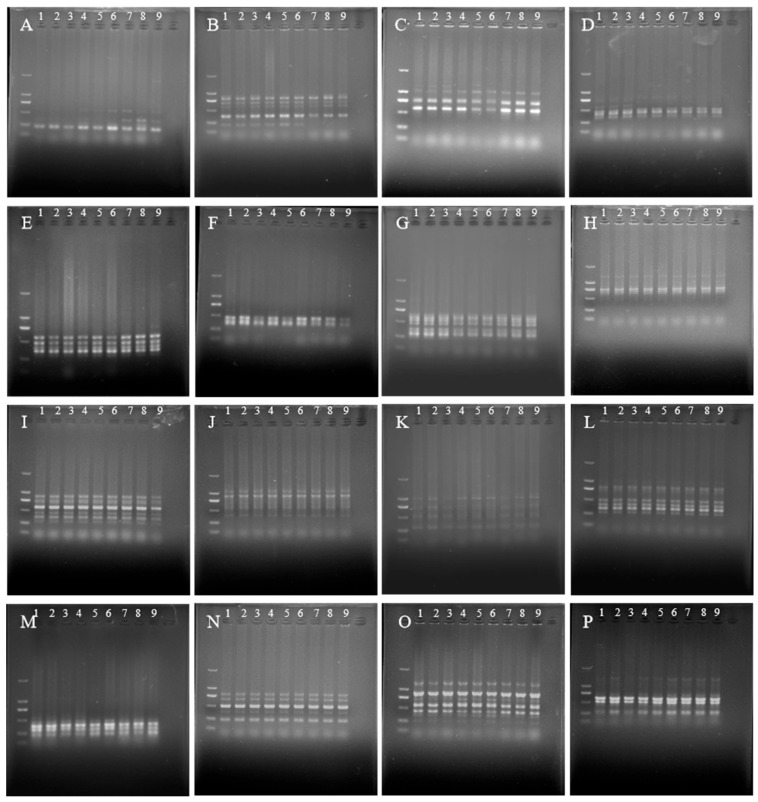
ISSR marker detection for ‘JD-12’ plants. (**A**): Primer 3A30, (**B**): Primer 3A37, (**C**): Primer 3A59, (**D**):Primer UBC895; (**E**): Primer 3A26; (**F**): Primer 3A50; (**G**): Primer UBC811; (**H**): Primer UBC814; (**I**): Primer UBC815; (**J**): Primer UBC820; (**K**): Primer UBC825; (**L**): Primer UBC835; (**M**): Primer UBC842; (**N**): Primer UBC843; (**O**): Primer UBC844; and (**P**): Primer UBC857. 1–3: Three independent Longshan *L. lancifolium* control samples; 4–6: three independent undoubled samples (‘JD-17’); and 7–9: three independent ‘JD-12’ samples. The first channel: Marker 2000.

To confirm the genetic variation in the ‘JD-12’ plants at the molecular level, 16 primers were selected for ISSR–PCR amplification, and polymorphic variations were detected. The results revealed that the amplified products obtained with four ISSR–PCR primers, 3A30, 3A37, 3A59, and UBC895 (Figure 10A–D), presented polymorphic differences between Langshan *L. lancifolium* control and ‘JD-12’ plants, while the amplified products using the other twelve primers were not significantly different (Figure 10E–P). On the basis of the amplification results, 246, 243, and 237 bands were detected from the control, ‘JD-17’ (undoubled line), and ‘JD-12’ plants, respectively. The average number of amplified bands per primer was 5.12, 5.06, and 4.93, respectively, and no variant bands were found in three independent samples of the control, ‘JD-17’, and ‘JD-12’ plants, indicating their relatively stable genetic characterization. However, compared with those of the control, the bands amplified from ‘JD-12’ using the ISSR primers were significantly different. A total of 82 bands were detected from the ‘JD-12’ plants, 5 of which were polymorphic, giving an ISSR band variation rate of 6.10%. In addition, only one band amplified by the UBC895 primer differed between ‘JD-17’ and the control, suggesting that the undoubled line was almost unchanged.

## 4. Discussion

The Longshan County of Hunan Province is one of the well-known origins of *L. lancifolium* in China. Therefore, Longshan *L. lancifolium* has become the pillar industry of the local economy. However, owing to the repeated use of older varieties and intensive planting over many years, the genetic characteristics of Longshan *L. lancifolium* have gradually deteriorated, which has led to a severe pest infestation and disease occurrence and has reduced the yield and quality. Thus, there is an urgent need to improve the genetic characteristics of Longshan *L. lancifolium* on the market by using new and/or high-quality varieties [7,10]. Polyploid lilies usually present excellent traits with enlarged organs and an enhanced resistance; thus, polyploidization has become an ideal method to improve multiple lily plant traits. However, there are no reports on the polyploid induction of Longshan *L. lancifolium* [13,14]. In recent decades, polyploid induction in lilies has depended mainly on using small bulblets, seeds, embryogenic calli/somatic embryos, and flower buds as materials [14]. Our research group induced *L. lancifolium* polyploids using bulblets originating from the Harbin area in Heilongjiang Province [23,25]. Herein, we chose Longshan *L. lancifolium* as the material, and the regenerated shoots were induced on MS media with different combinations of NAA and TDZ, and colchicine was used to induce the polyploidy in the regenerated shoots in vitro.

In this study, we applied four concentration gradients and three different treatment durations and found that the morphology of regenerated shoots changed significantly after 10 days of the colchicine treatment. With increasing concentrations of colchicine, the frequency of morphological shoot variants first increased but then decreased. A similar phenomenon was observed in previous studies, such as during the induction of polyploidy in the lily ‘Yelloween’ [29] and *L. tsingtauense* [30], which might be due to the strong toxicity of colchicine to lilies. In general, as the concentration of colchicine increased, the toxicity to the experimental materials increased, thus causing browning or death. Finally, the greatest mutagenic effect on the regenerated shoots of Longshan *L. lancifolium* was observed after the treatment with 0.10% colchicine for 48 h, at which time the frequency of morphological variants peaked (80.00%). The frequencies of morphological variants was significantly greater than those (54. 29%, 60.0%, and 58. 06%, respectively) in lilies with small bulblets [23,26,31] or (30.0% and 65. 57%) in lilies when seeds were used [32,33]. Therefore, it is possible that regenerated shoots could be more sensitive to colchicine, which may make achieving the expected mutation effect easier based on morphology screening. This could be why Wu et al. (2008) [34] and Yang et al. (2014) [35] used adventitious shoots or clustered shoots to induce polyploids of colored *Zantedeschia*.

FCM is a highly efficient tool for identifying the chromosome ploidy; it can quickly and accurately detect the nucleic acid content in cells and is often used for the ploidy analysis of many experimental samples [27]. In this study, FCM was used to analyze the chromosome ploidy of 41 Longshan *L. lancifolium* morphologically mutant plantlets, and two polyploids were found to have an induction rate of 4.88%. This induction rate was significantly lower than the mutation rate of somatic embryos induced by colchicine in *L. pumilum* (19.85%) [17] and was lower than the mutation rates of scales induced by colchicine in *L. distichum* Nakai (23.25%) and *L. cernuum* Komar (14.14%) [36]. This might be because Longshan *L. lancifolium* is a triploid plant. Excluding the differences in the responses of different lily species, the lower induction rate might be because the regenerated shoots were prone to morphological changes upon the colchicine treatment; thus, the plantlets that truly underwent chromosome doubling were difficult to select. Therefore, it may be better to develop alternative methods with a low toxicity and the ability to induce chromosome doubling, such as aminopurine or fluridone treatments for induction. However, which agents are more suitable for chromosome doubling in regenerated shoots remains to be further studied. In addition, morphological characterization would be affected by the phytohormone and environment conditions. And morphological screening alone was not sufficient for selecting polyploids in the early stage. Thus, morphological screening with other developed methods would be better for selecting polyploids in the near future.

During the colchicine-induced polyploidy, chimaerism is prone to occur because of uncoordinated cell division or the different positions of the cell cutting [17,29]. In this study, two polyploid plants were obtained, one of which was a chimeric polyploid (‘JD-37’), and the other of which was a homozygous polyploid (‘JD-12’). Chimeric plants are prone to degeneration in later cultivation processes; therefore, there are no reports of chimaerism in lily varieties except for the Asian lily group [37]. In this study, the homozygous polyploid ‘JD-12’ was selected, and its plant height was slightly reduced, its blade surface was rougher, its leaf color was a darker green, and its scales were significantly thicker. This morphology conforms to the basic traits of polyploid plants [19,38]. The microscopic observation revealed that the leaf epidermal cells and guard cells of ‘JD-12’ were significantly enlarged and that the stomatal density was reduced, indicating that this plant could be initially considered a polyploid plant [17,36]. A previous study revealed that the stomatal density of triploid plants was 24.73~48.84 mm^−2^, whereas that of tetraploids was 17.36~28.42 mm^−2^ [39]. The stomatal density of the control was 29.75 mm^−2^, which was in line with the stomatal density of triploids, whereas the stomatal density of ‘JD-12’ was 9.66 mm^−2^, which was significantly lower than that of the control and the tetraploids. This phenomenon indicated that ‘JD-12’ was a polyploid compared with the control and is consistent with the above FCM analysis. The reduced stomatal density might be more advantageous for ‘JD-12’ to adapt to the adversity environment. In addition, the stomatal densities of the control and ‘JD-12’ tissue-cultured plantlets were 34.00 and 17.47 mm^−2^, respectively, which were greater than those of the outdoor-grown plantlets (29.75 and 9.66 mm^−2^), indicating that the stomatal density may be closely regulated by environmental light and humidity.

Identifying variant plants by detecting molecular markers is a more reliable method than the identification by morphological observations [40,41]. The ISSR is a new DNA molecular marker technique developed on the basis of microsatellites that is characterized by clear amplification bands, a simple operation, and reproducible data. Thus, this method has been widely used for the authenticity identification and genetic variation analysis of *Lilium* polyploids [17,19,42]. In our experiments, the polymorphic bands in the ‘JD-12’ genome were amplified with 16 ISSR primers. Compared with the control, four ISSR primers yielded products with obvious changes in the amplification bands, indicating that the ‘JD-12’ plants exhibited genetic variations at the molecular level, with a variation rate of 6.10%. However, this mutant rate in ‘JD-12’ was lower than that of the colchicine-induced polyploids of *L. pumilum* and *L. davidii* var. *unicolor* (15.48% and 9.75%, respectively) [17] (Sun et al., 2018) and the No. 2 *L. leichtlinii* var. *Maximowiczii* mutant (18.99%) [28] (Fu et al., 2025). We suggested that this may account for the genomic information or/and chromosome ploidy of different lily species. In addition, the amplification bands of ‘JD-17’, an undoubled line, were almost unchanged related to the Langshan *L. lancifolium* control, suggesting that ‘JD-12’ was successfully induced by colchicine. Overall, we identified the polyploid ‘JD-12’ from Longshan *L. lancifolium* and further to investigated the characteristics of its adult plants, such as the bulb size, nutrient content, and pathogen resistance. Our data lay a foundation for the acquisition of high-quality and resistant varieties of Longshan *L. lancifolium*.

This study advances previous work by expanding polyploid induction efforts to a genetically and geographically distinct population (Longshan *L. lancifolium*) and by utilizing regenerated shoots as explants, rather than the more commonly used bulblets or seeds. These two aspects introduce important biological and methodological variations, reflected in differences in the colchicine response, variant frequency, and morphological outcomes. The successful induction and characterization of the polyploid line ‘JD-12’ provides the first detailed report of polyploid breeding in Longshan *L. lancifolium*, offering practical insights for cultivar improvement and reinforcing the significance of genotype-specific strategies in lily polyploidization.

## 5. Conclusions

In this study, regenerated shoots of Longshan *L. lancifolium* were induced, and polyploid plants were generated in vitro after soaking the shoots in colchicine. The results showed that MS medium supplemented with 0.2 mg/L of NAA and 0.4 mg/L of TDZ was suitable for the regeneration of Longshan *L. lancifolium* shoots. The greatest mutation effect was obtained by treating the shoots with the 0.10% colchicine for 48 h, resulting in an 80.00% frequency of morphological variants and a survival frequency of 86.67%. A homologous polyploid, ‘JD-12’, was obtained with a chromosome number of 68 in the root tip cells. Compared with the control, the ‘JD-12’ plantlets were slightly dwarfed, the leaf color was deeper, the surface was rougher, and the bulblet diameter and scales were thicker. The upper epidermal cells and guard cells in ‘JD-12’ were larger than those in the control, and the stomatal density was significantly lower. The identification via the ISSR marker revealed the genetic variation in ‘JD-12’, with a variation rate of 6.10%. These results lay a foundation for the polyploidization breeding of Longshan *L. lancifolium* from regenerated shoots and the cultivation of high-quality and resistant varieties.

## Figures and Tables

**Table 1:** Effects of different colchicine concentrations on regeneration shoots in Longshan *L. lancifolium*.

Colchicine Concentration (*w*/*v*)	Treatment Duration (h)	Treated Number	Survived Number	Survival Frequency (%)	Number of Morphological Variants	Frequency of Morphological Variants (%)
0.05%	24	30	29	96.67	16	53.33
	48	30	28	93.33	20	66.67
	72	30	24	80.00	15	50.00
0.10%	24	30	27	90.00	20	66.67
	48	30	26	86.67	24	80.00
	72	30	26	86.67	23	76.67
0.15%	24	30	28	93.33	21	70.00
	48	30	25	83.33	23	76.67
	72	30	23	76.67	20	66.67
0.20%	24	30	26	86.67	22	73.33
	48	30	27	90.00	17	56.67
	72	30	21	70.00	17	56.67

**Table 2:** Comparison of plant morphology in tissue-cultured Longshan *L. lancifolium* control and ‘JD-12’ plantlets.

Samples	Plant Height (cm)	Leaf Length (cm)	Leaf Width (cm)	Leaf Thickness (mm)	Bulblet Diameter (cm)	Scale Length (cm)	Scale Width (cm)	Scale Thickness (mm)
Control	7.53 ± 0.12 *	6.45 ± 0.13 **	0.23 ± 0.01 **	0.37 ± 0.00	0.48 ± 0.02	0.52 ± 0.02	0.28 ± 0.01	1.76 ± 0.07
‘JD-12’	7.01 ± 0.26	5.97 ± 0.11	0.17 ± 0.01	0.76 ± 0.00 **	0.60 ± 0.03 **	0.51 ± 0.01	0.30 ± 0.01	2.12 ± 0.10 **

Note: The data in the table are shown as the mean value ± standard error. The stars (* or **) indicate significant differences (*p* < 0.05 or *p* < 0.01). The same as below.

**Table 3:** Comparison of leaf epidermic cells and guard cells in Longshan *L. lancifolium* control and ‘JD-12’ plants in greenhouse.

Samples	Upper Epidermis Cell Length (μm)	Upper Epidermis Cell Width (μm)	Cell Length–Width Ratio	Guard Cell Length (μm)	Guard Cell Width (μm)	Stomatal Frequency (No.·mm^−2^)
Control	418.07 ± 7.87	81.75 ± 1.45	5.46 ± 0.18	90.73 ± 0.55	57.03 ± 0.25	29.75 ± 1.49 **
‘JD-12’	569.62 ± 20.33 **	91.12 ± 2.40 **	7.08 ± 0.37 **	146.53 ± 1.97 **	70.67 ± 0.76 **	9.66 ± 0.37

Note: The stars (**) indicate significant differences (*p* < 0.01).

## Data Availability

All the supporting data can be found as additional files along with this manuscript. Data are contained within the article and Supplementary Materials.

## Supplementary Materials

The following supporting information can be downloaded at https://www.mdpi.com/article/10.3390/plants14131987/s1: Table S1. The primers used in the ISSR marker analysis. Table S2. Effects of different combinations of thidiazuron (TDZ) and naphthaleneacetic acid (NAA) on shoot induction. Table S3. Comparison of leaf epidermal cells, guard cells, and stomata in tissue-cultured plantlets.

## References

[1] Wang F.Z., Tang J. (1980). Flora of China.

[2] Zhao X.Y., Wang W.H. (2017). Status and Development Proposals of Lily Industry in China[M]//Ming J, Yuan SX. Papers (Abstracts) of the 12th Symposium of Chinese Society of Flower Bulbs.

[3] Luo C.Y., Yuan Z.T., Liu H.J., Yuan Y., Zheng S.X. (2024). Research progress in the utilization of medicinal lily germplasm resources. Hunan Agric. Sci..

[4] Yang L.P., Fu Y.Y. (2018). Research on the Utilization of Lily Resources in China.

[5] Yang L.P., Song X.H. (2013). Tissue culture system construction of *Lilium lancifolium* Thunb. J. Agric. Univ. Hebei.

[6] Wang Y.L., He W.R., Wang S.H., Hu L.Q., Yu X.L., Li J.G. (2023). Industry status and development direction of Longshan medicinal lily. Hunan Agric. Sci..

[7] Pan Q.P., Zhou R.B., He Y.S., Luo Y.L. (2003). Investigation of lily bulb implantation garden in Longshan County. Hunan Guid. J. TCM.

[8] Yang Q. (2014). Lily Cultivation, Yield and Quality Improving Policy and Technology in Longshan County. Master’s Thesis.

[9] Lei L.H. (2015). Antioxidant Capacity and Lung Cancer Cell Rejection Characterization of *Lilium lancifolium* Bulbs from Different Populations. Master’s Thesis.

[10] Wang C.L., Yuan C.Z., Chen H.X., Jiang H. (2015). Detection of CMV infecting on Longshan *Lilium tigrinum*. Tianjin Agric. Sci..

[11] Jiang H., Yuan C.Z., Chen H.X. (2014). Detection of LSV infecting Longshan *Lilium tigrinum*. Tianjin Agric. Sci..

[12] Fu Y.Y., Yang L.P., Gao H.H., Xu W.J., Lei M.Y., Wang A.X. (2020). Virus detection of *Lilium lancifolium* Thunb. in Chongqing and study on its detoxification technology. Acta Agric. Boreali-Occident. Sin..

[13] Hu Y., Li H.G., Lei X.Y., Zhou Y.J., Zhang Y., Li L.H. (2022). Study on mutagenic effects of ^60^Co-γray and chemical mutagen on *Lilium lancifolium*. Acta Agric. Jiangxi.

[14] Han X., Yang L.Y., Chen M.M., Li X., Yang Y.Y., Zhang Y.C. (2024). Research progress of the techniques applicable in lily breeding. J. Plant Genet. Resour..

[15] Yamagishi M., Jitsuyama Y., Hoshino Y. (2023). Agronomic performance in tetraploid *Lilium leichtlinii*: Larger flowers and earlier flowering. Euphytica.

[16] Wu X.J., Yang L.P., Chen M. (2016). In vitro polyploid induction of *Lilium callosum*. Guizhou Agric. Sci..

[17] Sun H.M., Fu L.L., Wang Z.P., Gai M.Z., Wang C.X. (2018). Polyploidy induction and identification of *Lilium pumilum* and *Lilium davidii* var. *unicolor* based on somatic embryogenesis. Acta Hortic. Sin..

[18] Wang Y.T., Zhang Y.Q., Yang Q.J. (2019). Effect of colchicine on polyploid induction of *Lilium* concolor seeds. Guizhou Agric. Sci..

[19] Fu Y.Y., Cai Li Li F.Y., Yang W.J., Xu W.J., Jiang S.J., Yang L.P. (2024). Induction and molecular and cellular identification of polyploid *Lilium davidii* var. unicolor. Acta Pratacult. Sin..

[20] Wang Z.G., Yin D.S., Zhang H.H., Wu T.T., Pan B.S., Yuan X.F. (2014). Research progress on genetic breeding of *Lilium lancifolium* Thumb. Hortic. Seed.

[21] Shu K., Li J.H., Chen L., Fu H.Y., Cai W., Liu L.C., Li Y.F. (2022). Distant hybridization of *Lilium lancifolium* Thunb. and identification of its hybrid progeny. J. Hunan Agric. Univ. (Nat. Sci.).

[22] Tang Y.C., Yang P.P., He G.R., Cao Y.W., Liu Y.J., Xu L.F., Ming J. (2022). ITS sequence analysis used for parent selection in *Lilium lancifolium* Thunb. cross-breeding. J. Appl. Res. Med. Aromat. Plants.

[23] Chen A., Yang L.P., Tan Y., Peng C.T., Tang B. (2014). Study on polyploid induction of *Lilium lancifolium* in vitro with colchicine treatment. J. Plant Genet. Resour..

[24] Lei M.Y., Yang L.P., Yang T.J., Fu Y.Y., Han L., Quan J., Pu S.C. (2020). Cultivation of a new polyploid *Lilium lancifolium* variety ‘Yu Baihe 1’. Mol. Plant Breed..

[25] Fu Y.Y., Yi D.Y., Yang X.M., Cai L., Liang Y.H., Lei M.Y., Yang L.P. (2022). Analysis of morphological characteristics and genetic variation in a new germplasm *Lilium lancifolium* JD-h-15. Biotechnol. Bull..

[26] Fu Y.Y., Liang X.M., Zhang H., Cheng S.Y., Li A.Q., Liao M.J., Tan L., Yang L.P., Qi X.Y. (2024). Establishment of an efficient regeneration system and in vitro polyploid induction based on the bulblet centre in *Lilium rosthornii* Diels. In Vitro Cell. Dev. Biol.-Plant.

[27] Chen M.M., Zhou Y., Sun Y.J., Li X., Sun J.J. (2018). Polyploidy induction of *Lilium* spp. by colchicine and ploidy identification by flow cytometry. Acta Agric. Shanghai.

[28] Fu Y.Y., Ren J.J., Guo R.Y., Lu X.X., Lei Y.X., Xu W.J. (2025). In vitro induction and molecular and cellular identification of mutant plants of *Lilium leichtlinii* var. *Maximowiczii* (Regel) Baker by colchicine. Ornam. Plant Res..

[29] Wu Q.Q., Hu X.J., Cui W., Yang L., Shi L.J., Xu H.J., Ban T.T., Xia J.F. (2019). Study on ployploid induction of lily Yelloween by colchicine. Seeds.

[30] Zhang J.F., Liu Q.H., Wang K.L., Liu Q.C., Sun Y. (2009). Tetraploid induction of *Lilium tsingtauense* by colchicine. J. Nucl. Agric. Sci..

[31] Liu J., Zhao Q.F., Ding L. (2011). Polyploid induction and indentification of *Lilium davidii* var. *unicolor* (Hoog) Cotton. North. Hortic..

[32] Yang Y.J., Ge B.B., Wei Q., Gao J.P., Hong B. (2013). Colchicines-induced polyploid plants and identification in *Lilium pumilum* DC. J. China Agric. Univ..

[33] Zhang X.Q., Wang L.J., Cao Q.Z., Jia G.X. (2017). Polyploidy induction and identification in *Lilium concolor* var. pulchellum. J. Beijing For. Univ..

[34] Wu H.Z., Zhang X.Q., Zheng S.X., Shi J.F., Bi Y.F. (2008). Polyploid induction of coloured *Zantedeschia aethiopica*. Acta Hortic. Sin..

[35] Yang H., Gou H., Zhou D., Du T., Wu H.Z. (2014). Studies on polyploid induction of different varieties of the colored Zantedeschia hybrid. J. Yunnan Agric. Univ..

[36] Fu L.L., Zhu Y.Y., Li M., Wang C.X., Sun H.M. (2019). Autopolyploid induction via somatic embryogenesis in *Lilium distichum* Nakai and *Lilium cernuum* Komar. Plant Cell Tissue Organ Cult. (PCTOC).

[37] Okazaki K., Hane Y. (2005). Comparison of diploid and chimeric forms (4×/2×) of Asiatic hybrid lilies (*Lilium* spp.) under natural and early forcing culture. N. Z. J. Exp. Agric..

[38] Wang L.Y., Jing R.Y. (2008). Tetraploid induction of *Lilium davidii* var. *unicolor* through colchicine treatment. J. Nucl. Agric. Sci..

[39] Wang M.L., Li X.T., Wang W.H., Du F. (2021). Ploidy identification of 100 lily accessions based on stomatal characteristics. Acta Agric. Jiangxi.

[40] Mallick P., Chattaraj S., Sikdar S.R. (2017). Molecular characterizations of somatic hybrids developed between *Pleurotus florida* and *Lentinus squarrosulus* through inter-simple sequence repeat markers and sequencing of ribosomal RNA-ITS gene. Biotechnology.

[41] Mcgregor C.E., Lambert C.A., Greyling M.M., Louw J.H., Warnich L. (2000). A comparative assessment of DNA fingerprinting techniques (RAPD, ISSR, AFLP and SSR) in tetraploid potato (*Solanum tuberosum* L.) germplasm. Euphytica.

[42] Zhang Z.J., Zheng Z.W., Zheng S.X., Liao X.S., Song Z.W., Song R., Lin Q.D. (2022). Cultivation and identification of allotriploid lily. Mol. Plant Breed..

