# Supplementary material for: In Vitro Polyploidy Induction of Longshan Lilium lancifolium from Regenerated Shoots and Morphological and Molecular Characterization
Source: Plants (Basel). 2025 Jun 29;14(13):1987. doi: 10.3390/plants14131987 (PMC12251911; doi:10.3390/plants14131987)
Supplement: Supplementary file 1 [file plants-14-01987-s001.zip › plants-3681622-supplementary.pdf]

Table S1. The primers used in the ISSR marker analysis.

Primers	Sequences (5’ to 3’)
3A26	CTCTCTCTCTCTCTGCA
3A30	CTCTCTCTCTCTCTGAA
3A37	CACACACACACACATGA
3A50	CACACACACACACAAGT
3A59	CTCTCTCTCTCTCTGTG
UBC811	GAGAGAGAGAGAGAGAC
UBC814	CTCTCTCTCTCTCTCTA
UBC815	CTCTCTCTCTCTCTCTG
UBC820	GTGTGTGTGTGTGTGTC
UBC825	ACACACACACACACACT
UBC835	AGAGAGAGAGAGAGAGYC
UBC842	GAGAGAGAGAGAGAGAYG
UBC843	CTCTCTCTCTCTCTCTRA
UBC844	CTCTCTCTCTCTCTCTRC
UBC857	ACACACACACACACACYG
UBC895	AGAGTTGGTAGCTCTTGATC

Table S2. Effects of different combinations of thidiazuron (TDZ) and naphthaleneacetic acid (NAA) on shoot induction.

MS/ (mg·L^{-1})		Scale number	Number of shooting scales	Total shooting number	Scale shooting rate (%)	Scale shooting coefficient
NAA	TDZ					
0.2	0	30.00	5.33 ± 1.45 b	8 ± 1.15 b	17.78 ± 4.84 b	0.26 ± 0.03 b
0.2	0.2	30.00	9.67 ± 3.18 ab	14.67 ± 4.70 ab	32.22 ± 10.59 ab	0.49 ± 0.16 ab
0.2	0.4	30.00	18.33 ± 2.60 a	29.33 ± 7.05 a	61.11 ± 8.67 a	0.97 ± 0.23 a
0.2	0.8	30.00	9.67 ± 3.48 ab	16.33 ± 5.36 ab	32.22 ± 11.60 ab	0.54 ± 0.18 ab
0.2	1.0	30.00	10.00 ± 2.51 ab	16.67 ± 5.81 ab	33.33 ± 8.38 ab	0.56 ± 0.19 ab

Table S3. Comparison of leaf epidermal cells, guard cells and stomata in tissue-cultured plantlets.

Samples	Upper epidermis cell length (μm)	Upper epidermis cell width (μm)	Cell length-width ratio	Guard cell length (μm)	Guard cell width (μm)	Stomatal frequency (No.·mm^{-2})
Control	297.75±3.95	58.72±0.91	5.28±0.12	86.35±0.31	67.95±0.45**	34.00±1.16**
'JD-12'	453.54±6.13**	74.75±1.36**	6.41±0.15**	95.02±0.71**	56.08±0.82	17.47±1.11
